# Development of a needle trap device packed with modified PAF-6-MNPs for sampling and analysis of polycyclic aromatic compounds in air

**DOI:** 10.1039/d4ra01651c

**Published:** 2024-06-10

**Authors:** Mobina Hashemi, Abdulrahman Bahrami, Farshid Ghorbani-Shahna, Abas Afkhami, Maryam Farhadian, Ali Poormohamadi

**Affiliations:** a Center of Excellence for Occupational Health, Occupational Health and Safety Research Center, School of Public Health, Hamadan University of Medical Sciences Hamadan Iran bahrami@umsha.ac.ir; b Department of Chemistry, Bu-Ali-Sina University Hamedan Iran; c Department of Biostatistics, School of Public Health and Research Center for Health Sciences, Hamadan University of Medical Sciences Hamadan Iran

## Abstract

The aim of this study was to develop a new method for sampling and analyzing polycyclic aromatic hydrocarbons in the air. This was achieved by utilizing a needle trap device packed with a modified porous aromatic framework coated with magnetic nanoparticles (PAF-6-MNPs). The modified adsorbent underwent qualitative evaluation using Fourier-transform infrared spectroscopy and X-ray diffraction, as well as scanning and transmission electron microscopy. The optimal conditions for sampling polycyclic aromatic hydrocarbons compounds were determined using a dynamic atmosphere chamber. The method was validated by taking various samples from the standard chamber, and then analyzed under different environmental sampling conditions using a gas chromatography device. The limit of detection (LOD) and limit of quantification (LOQ) values for the analytes of interest, including naphthalene, anthracene, and pyrene, ranged from 0.0034–0.0051 and 0.010–0.015 μg L^−1^, respectively. Also, the repeatability and reproducibility of the method expressed as relative standard deviation, for the mentioned analyses were found to be in the range of 17.8–20.5% and 20–22.9%. The results indicated that over a 20 day storage period (with the needle trap device containing the analytes of interest kept in the refrigerator), there was no significant decrease in the amount of analytes compared to the initial amount. These findings suggest that, the needle trap packed with the proposed adsorbent offers a reliable, highly-sensitive, easy-to-use, and cost-effective method for sampling polycyclic aromatic hydrocarbons in the air compared to the conventional method recommended by the National Institute of Occupational Safety and Health (NIOSH), method 5515.

## Abstract 2

The aim of this study was to develop a new method for sampling and analyzing polycyclic aromatic hydrocarbons in the air.

## Graphical abstract

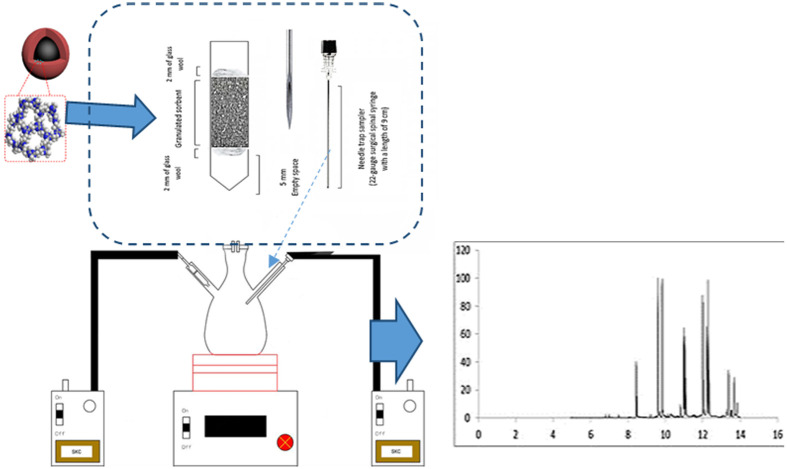

## 1. Introduction

Polycyclic Aromatic Hydrocarbons (PAHs) are white or light yellow solids with low solubility in water^1^ and high melting and boiling points. These compounds are mainly produced during the incomplete combustion of organic materials such as coal, oil, gasoline, and wood.^2^ The main natural and man-made sources of PAHs include the fires of forests and pastures, fossil fuels combustion, vehicle exhaust emissions, and industrial workplace air emissions.^1–4^ The International Agency for Research on Cancer (IARC) has classified benzopyrene into group A of carcinogenic compounds.^5–8^ Moreover, PAHs cause thrombotic effects in individuals with coronary heart disease, and acute exposure is associated with symptoms such as burning of the eyes, nausea, vomiting, and diarrhea.^9–12^

Personal sampling from worker's breathing zone in workplaces and environmental sampling are crucial methods for evaluating PAH exposure. The most common method for identifying PAHs compounds is based on the use of surface absorbent tubes and filters with personal sampling pumps. However, the conventional methods have low sensitivity due to the dissolution of trapped analytes in a solvent during sample preparation, rendering PAHs undetectable at low concentrations. On the other hand, the occupational exposure limit of this pollutant has been decreased in the new updates of the standards. Therefore, the conventional methods with their low sensitivity and complex extraction procedures in the preparation stage, need to be replaced with new micro-extraction sampling methods to overcome these limitations.

Needle Trap Device (NTD), as a novel sampling and analysis methods, is based on the solid phase extraction method,^13–15^ akin to Solid Phase Micro-Extraction (SPME). However, unlike SPME method, the NTD is considered an active sampling method, which is based on using a personal sampling pump.^13,16^ NTD involves packing a specific amount of absorbent inside a small needle, enabling the trapping and qualitative/quantitative identification of analytes in air samples. NTDs are inexpensive, robust, reusable, and suitable for single-step sampling^13–22^ and analysis of Volatile Organic Compounds (VOCs) from different matrices. Their user-friendly operation and ability to detect low concentrations of organic compounds have garnered significant attention from scientists worldwide.^16,17^ Notably, NTDs can serve as a cost-effective alternative to thermal desorption apparatus, which is expensive and less readily available.

Metal–Organic Frameworks (MOFs) adsorbents have recently been employed for measuring PAHs compounds in air.^21^ Despite their successful application in preparing and analyzing air samples, MOFs have several limitations, including lower chemical stability, weaker coordination interaction, and fragility at temperatures above 300 °C. In contrast, Porous Organic Frameworks (POFs) are systematically constructed from organic monomers, comprising lightweight non-metallic elements (*e.g.*, C, H, N, B, O, and Si) bonded by strong covalent bonds. Among POFs, Porous Aromatic Frameworks (PAFs),^23^ stand out due to their unique molecular structures, simple and regular configurations, high surface area, homogeneous and porous distribution, high thermal stability, special hydrophobic–hydrophilic nature, and high charge. PAFs have been widely employed for sampling various compounds in different matrices such as water, soil, biological tissues, and plant tissues using various sampling methods.^24–26^ However, to date, no study has explored the application of NTD packed with PAF-6 for single step sampling and analysis of pollutants from air.

PAF-6 is synthesized through the reaction between piperazine monomers and cyanuric chloride. The reaction temperature is controlled to achieve one-step polymerization, resulting in an adsorbent for NTD characterized by biocompatibility, non-toxicity, chemical stability, and porosity. PAF-6 is expected to serve as a valuable adsorbent for NTD, capable of adsorbing a wide range of analytes with different polarities.^27–29^

Our review, did not reveal any study analyzing air pollutants using the NTD packed with Porous Aromatic Framework-coated Magnetic Nanoparticles (PAF-6-MNPs) as an adsorbent. In this study, the PAF-6-MNPs adsorbent was first synthesized and modified in the laboratory, and then packed inside the spinal needle to create the desired NTD. Subsequently, an NTD-PAF-6-MNPs sampler was employed to sample and analyze gas-phase PAH compounds (anthracene, pyrene, and naphthalene) from the air. Finally, the performance of the proposed method was compared with the standard method recommended by the National Institute of Occupational Safety and Health (NIOSH-5515).^30^ The sampling and analysis conditions were optimized under laboratory conditions, and the method was validated and developed for the target analytes.

## 2. Experimental

### 2.1 Chemical materials

Naphthalene (C_10_H_10_, 99%), anthracene (C_14_H_10_, 99%), pyrene (C_16_H_10_, 99%), dioxane (98%), cyanuric chloride (99%), dimethylethanolamine (99.9%), toluene (99.8%), piperazine, acetone (99.8%), ammonia solution (28 wt%), and HPLC grade methanol (MeOH) were purchased from Sigma-Aldrich. All solutions were prepared with distilled water at room temperature. Ultrapure water was used for the experiments by Milli-Q system (Millipore, USA). XAD-2 Sorbent Tubes were purchased from SKC (USA). Nitrogen with high purity (99.99%) was obtained from Roham Co (Tehran, Iran).

### 2.2 Apparatus

In this study, the Gas Chromatography (GC) analysis was performed using an Agilent 7890B device equipped with a Fame Ionization Detector (FID) and an HP-5 capillary column (30 m, 0.32 mm diameter, and 0.25 μm film thickness) to analyze PAHs compounds from the air. The GC was set at a split ratio of 1 : 8 and the column flow rate of 1.5 mL min^−1^ for the analysis of PAHs according to NIOSH method^30^ and split less for the analysis with NTD-PAF-6 MNPs. High-purity nitrogen gas (N_2_; 99.99%) was used as the carrier gas. The column temperature program started at 80 °C and increased to 180 °C at a rate of 32 °C min^−1^ followed by an increase of 300 °C at a rate of 15 °C min^−1^, with a constant hold at 300 °C for 2 minutes. The total program duration was 13.1 min. The FID temperature was set at 325 °C. A personal sampling pump (SKC 222 series, PA, USA) with a sampling flow rate of 1–200 mL min^−1^ was used to sampling with both NIOSH and NTD method. The flow rate of sorbent tube (Amberlite XAD-2) according to NIOSH method^30^ was set to 50–100 mL min^−1^, while for the NTD method (packed with PAF-6), it ranged from 0.3–1.3 mL min^−1^.

### 2.3 Sorbent synthesis

We have made changes to improve the synthesis of the adsorbent and have simplified its process as follows; first, 1.5 g of cyanuric chloride, and 1.8 mL of dimethylethanolamine were added to a flask containing 100 mL dioxane. The flask was put in a container filled with ice at a temperature of 0 °C. Subsequently, 1 g of piperazine was slowly dissolved in 40 mL of dioxane and added dropwise to the flask inside the container. The solution in the Erlenmeyer flask was maintained at 0 °C for 4 h. Following this, the solution was subjected to ultrasonication for 30 minutes until complete homogeneity was achieved. Next, the solution was placed under stream of nitrogen gas at 50 °C for 4 h to evaporate the liquid phase. The resulting solid was then placed in an oven at a temperature of 95 °C for 24 h. The solid product was subsequently washed multiple times with acetone, methanol, deionized water, and tetrahydrofuran solvents. The resultant solution was filtered using filter paper, and the adsorbent was oven-dried once more. In the second step of the adsorbent synthesis, 0.2 g of iron bisulfate was dissolved in a solution of water and hydrazine in a 3 : 1 ratio. The solution was ultrasonicated for 10 min until it attained a green hue.^31^ Finally, 0.1 g of PAF-6 was added to the solution, which was then subjected to ultrasonication. The solution PH was adjusted to 11 using an ammonia solution, and the solution was refluxed for 2 hours. Finally, the adsorbent was separated from the solution using a magnet and dried.^32^

### 2.4 Sampling chamber

**Fig. 1:**
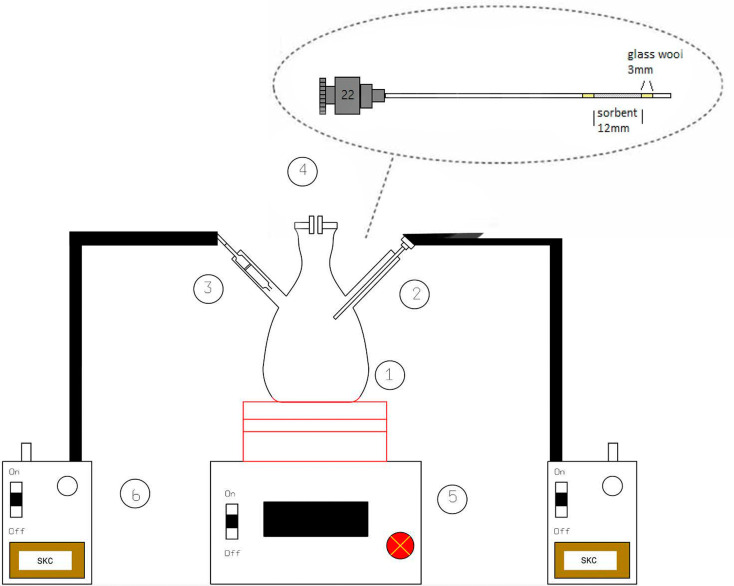
Schematic diagram of sampling chamber ((1) glass chamber, (2) NTD sampling, (3) XAD-2 sorbent tubed, (4) inlet/outlet control, (5) heater, (6) low flow pump).

**Fig. 2:**
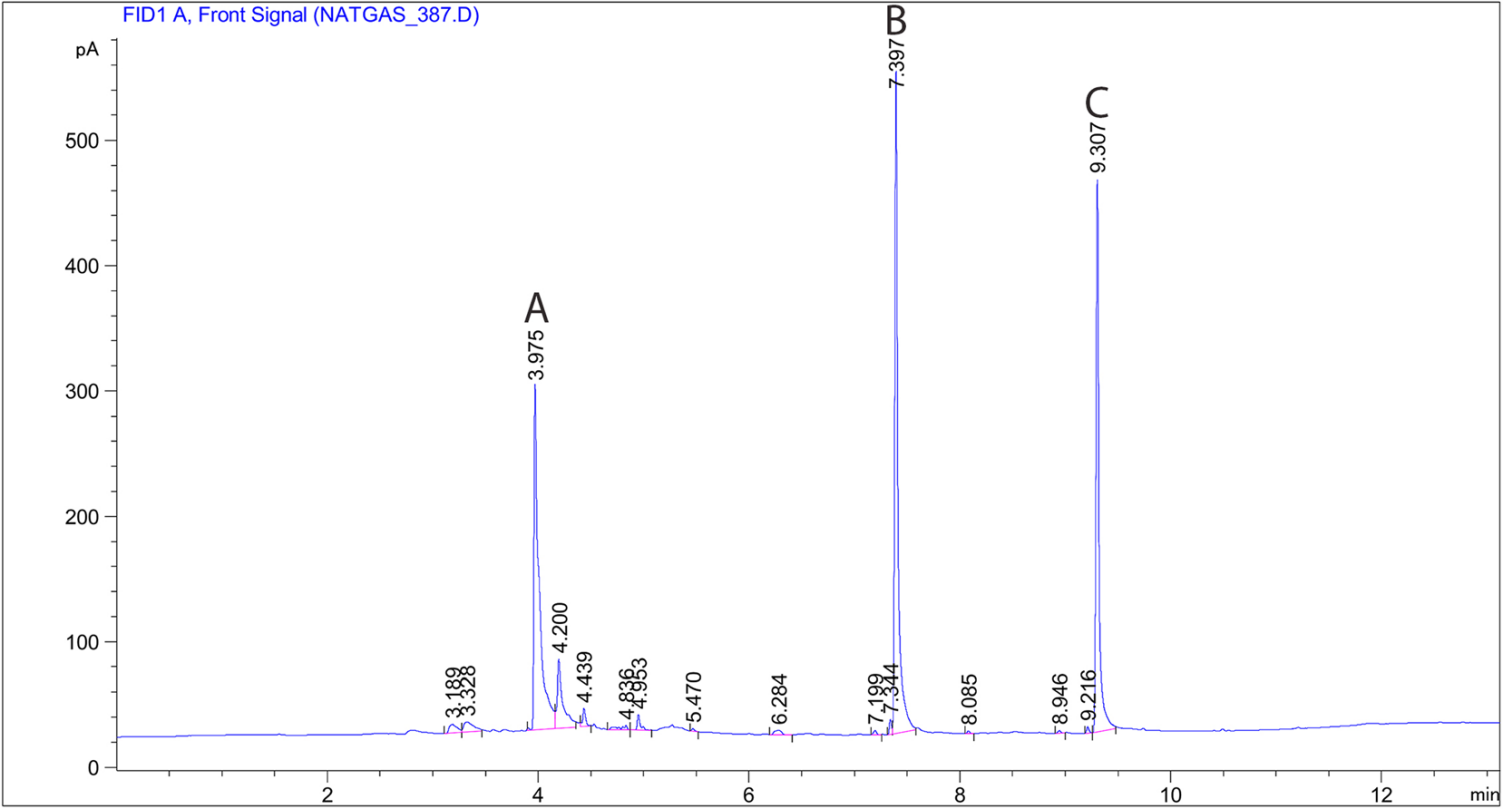
Chromatogram obtained with NTD-PAF-6 MNPs sampling: (A) naphthalene, (B) anthracene and (C) pyrene.

In the present study, a modified large glass Erlenmeyer flask (as depicted in Fig. 1) served as the sampling chamber for controlling sampling conditions. The chamber was also utilized for preparing dynamic standard samples with NTD to establish a calibration curve for determining the concentrations of PAHs. The sampling chamber featured of three outlets: one for sampling with the proposed NTD, another for sampling with the XAD-2 sorbent tube accordance with the NIOSH-5515 method,^30^ and an air inlet to prevent air streaming inside the chamber. Side by side sampling using both methods was conducted to determine the concentrations of anthracene, pyrene, and naphthalene in the sampling chamber. Additionally, various concentrations were prepared by introducing different amounts of each PAHs into the glass chamber and then placing the chamber on a heater at 80 °C for 20 minute. Subsequently, the chamber door was opened, and the samples were collected using a low flow sampling pump. Fig. 2 illustrates a chromatogram sample obtained from the sampling chamber.

### 2.5 NTD preparation

In this study, as illustrated in Fig. 1, a 22-gauge surgical spinal syringe with a length of 9 cm (outer diameter = 0.71 mm and inner diameter = 0.39 mm) served as the NTD body for inserting the absorbent. Initially, 2 mm of glass wool was positioned at the beginning of the needle of the syringe to secure the absorbent. Subsequently, a 1 cm space within the syringe was filled with the granulated sorbent (using standard ASTM sieves with a mesh size of 40–60). Finally, 2 mm of glass wool was inserted at the end of the needle to anchor the sorbent inside the needle (with 5 mm of its tip was empty).

The airflow rate passing through the NTD packed with the proposed sorbent was determined by connecting the initial part of the prepared NTD to the end of a pipette (with a volume of 1 mL) *via* a completely sealed silicone hose, which has then attached to the inlet of sampling pump. Next, the flow rate of the packed needle was calculated based on the movement time of the bubble in the pipette. Finally, after preparing NTDs, those with a flow rate of 0.8 ± 0.5 mL min^−1^ were selected for sampling, while NTDs with a flow rate outside the desired range were excluded from the study.

To prepare the predetermined concentrations, specific amounts of anthracene, naphthalene, and pyrene were poured into the glass sampling chamber (Fig. 1). The glass chamber was then placed on the heater at a temperature of 80 °C. After sampling for 30 minutes and injecting the NTDs containing the analytes of interest, they were introduced into the injection port of the GC device to desorb the analytes. The optimized temperature in the injection part of GC combined with passing the inert gas through the needle trap, facilitated the transfer of compounds to the GC column.

### 2.6 Determination of optimal desorption time and temperature

The full factorial analysis method was applied to design the NTD experiments aiming to examine the impact of desorption temperature and desorption time in the injection port of the GC device. For this purpose, 9 tests were conducted at three temperature levels of 280, 310, and 380 °C and three injection time levels of 2, 4, and 6 minutes. The data were input into Design-Expert software (version 7), and analyzed using the equation:
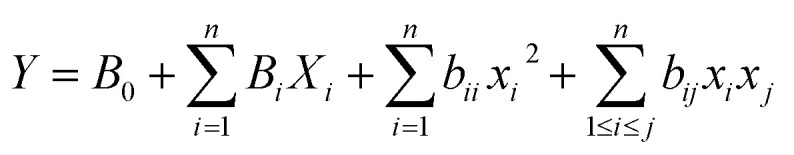
where, *Y* represents the predicted responses, *B*_0_ denotes the model's constant, *B*_*i*_ signifies the variable coefficient, and *x*, *n*, *b*_*ii*_, and *b*_*ij*_ stand for the coded variables, the number of variables, the quadratic variable coefficient, and the variable interaction coefficient, respectively. The coefficients were derived from experimental tests and regression analysis. Additionally, the fitting of response levels and optimization of variables were carried out using analysis of variance (ANOVA). This research was conducted in a completely random manner with 9 runs and 2 blocks (over two days).

### 2.7 Breakthrough volume investigation

In NTD sampling, breakthrough volume (BTV) is influenced by factors such as the length of the adsorbent packed inside the NTD, type of adsorbent, used, and the affinity and volatility of the analytes of interest. In this study, the determination BTV involved, pouring different amounts of PAHs into the sampling environment of the glass chamber at five levels to create specific concentrations. Subsequently, sampling with the proposed NTD was carried out and the samples were analyzed using the GC device.

### 2.8 Carry-over effect

The memory effect in NTD refers to the concentration of analyte that remains on the adsorbent after sample desorption, potentially interfering with subsequent uses of the NTDs. Given that NTD is a reusable sampling and analysis method, the carry-over effect can significantly impact method development. To assess this, the concentration inside the sampling chamber was adjusted to 2 to 3 μg L^−1^ (10 times more than PELs). Following the determination of optimal conditions and analysis desired PAHs compounds with a prepared NTD, the NTD was re-injected (without sampling) to quantify the remaining amount of PAHs in the NTD. The presence of peak area responses corresponding to the target analytes indicates the presence of a memory effect.

### 2.9 Method validation

#### 2.9.1 Limit of detection and limit of quantification and linear dynamic range

The efficiency of the NTD sampling method with PAF-6 MNPs in the sampling and analysis of PAHs in air samples was validated by evaluating the Limit of Detection (LOD), Limit of Quantification (LOQ), and Linear Dynamic Range (LDR). LDR was determined using the NTD calibration line equation. The LOD and LOQ were calculated by the following equation;LOD = 3.3*σ*/*S*,LOQ = 10*σ*/*S*where: *S* is the slope of the calibration curve and *σ* is the standard deviation of the blank needle trap.

#### 2.9.2 Accuracy

To assess the accuracy of the current method, sampling from the chamber was conducted simultaneously using XAD-2 sorbent (following NIOSH-5515 guidelines) and NTD-PAF-6-MNPs. Subsequently, after analysis using gas chromatography, the correlation of the data was evaluated.

#### 2.9.3 Repeatability & reproducibility

Relative Standard Deviation (RSD) was used to determine the repeatability and reproducibility of the method introduced. Repeatability was determined by sampling a specific amount of PAHs using the prepared NTDs. The concentration of PAH in sampling chamber ranged from 0.05 to 0.5 μg L^−1^ (close to 0.2 mg m^−3^; permissible exposure limit (PEL)^33^ recommended by Occupational Safety and Health Administration).

In this study, reproducibility was determined using different NTDs, followed by sampling and analysis of the pilot medium at the same time and concentration. Subsequently, the standard deviations of the experimental results were estimated.

### 2.10 Storage time

The storage capability of the newly proposed NTD in retaining the analytes of interest after sampling was evaluated by collecting 10 samples at the same concentration (0.05 to 0.5 μg L^−1^) and time from the sampling glass chamber. Following sampling, two NTDs were promptly analyzed, and both sides of the remaining NTDs were covered with silicone caps and stored at a temperature of 25 °C. These NTDs, post-sampling under the specified conditions, were retained and analyzed at time intervals of 10, 20, 30, and 40 days for each sample.

## 3. Results & discussion

### 3.1 Characterization of PAF-6-MNPs

**Fig. 3:**
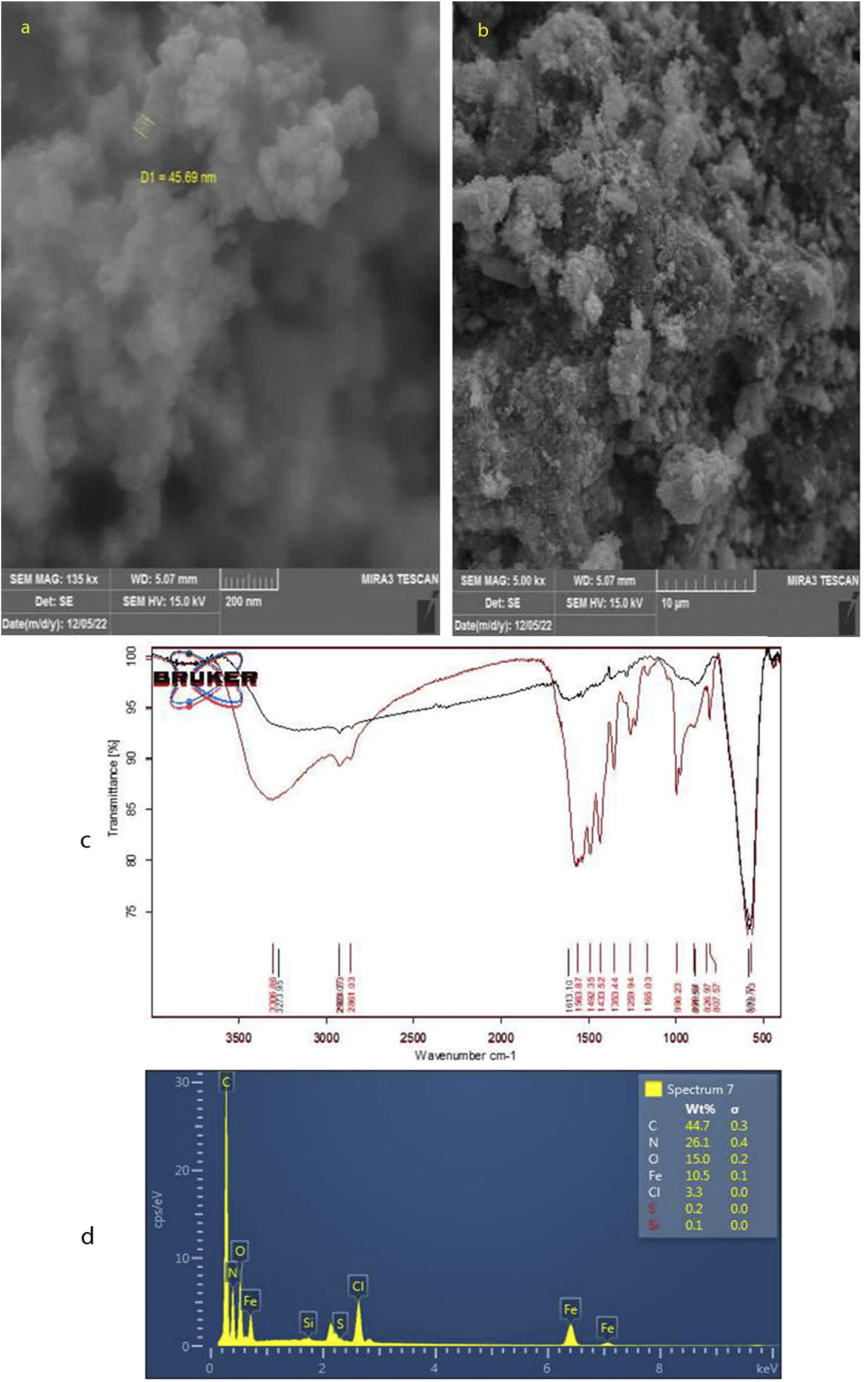
(a) and (b) SEM images of PAF-6 MNPs sorbent at different magnifications. (c) FTIR spectrum, and (d) the EDS pattern of PAF-6 MNPs sorbent.

According to Fig. 3a and b, the nucleus and shell structures exhibit porous micro-structure and agglomerates of irregular shapes with an approximate diameter of 50 nm. The properties of PAF-6 MNP are illustrated by the absorbent spectrum in Fig. 3c, which demonstrates the highest absorption in the range of 560 cm^−1^, representing the Fe–O bond (the black curve corresponds to the FTIR spectrum of iron oxide nanoparticles). The spectrum of PAF exhibited absorption peaks at 1492 cm^−1^ and 1165 cm^−1^, which are specific to triazine rings. These peaks correspond to the stretching vibration of C=N, suggesting that PAF6 is incorporated into magnetic nanoparticles. The maximum absorption at around 1000 cm^−1^ is indicative of the asymmetric stretching vibration of Si–O–Si, suggesting successful coverage of SiO_2_ on the Fe_3_O_4_ surface. In the laboratory, magnetic particles dispersed in the solution are swiftly collected using a magnet, highlighting the strong magnetic response of PAF-6 MNPs for magnetic separation.^31^ The chemical composition of the obtained sorbent was examined by EDS. As shown in Fig. 3d, the highest weight percentages were attributed to carbon, nitrogen, oxygen and iron, respectively, supporting the coating of Fe_3_O_4_ with the PAF layer.

### 3.2 Desorption parameters

**Fig. 4:**
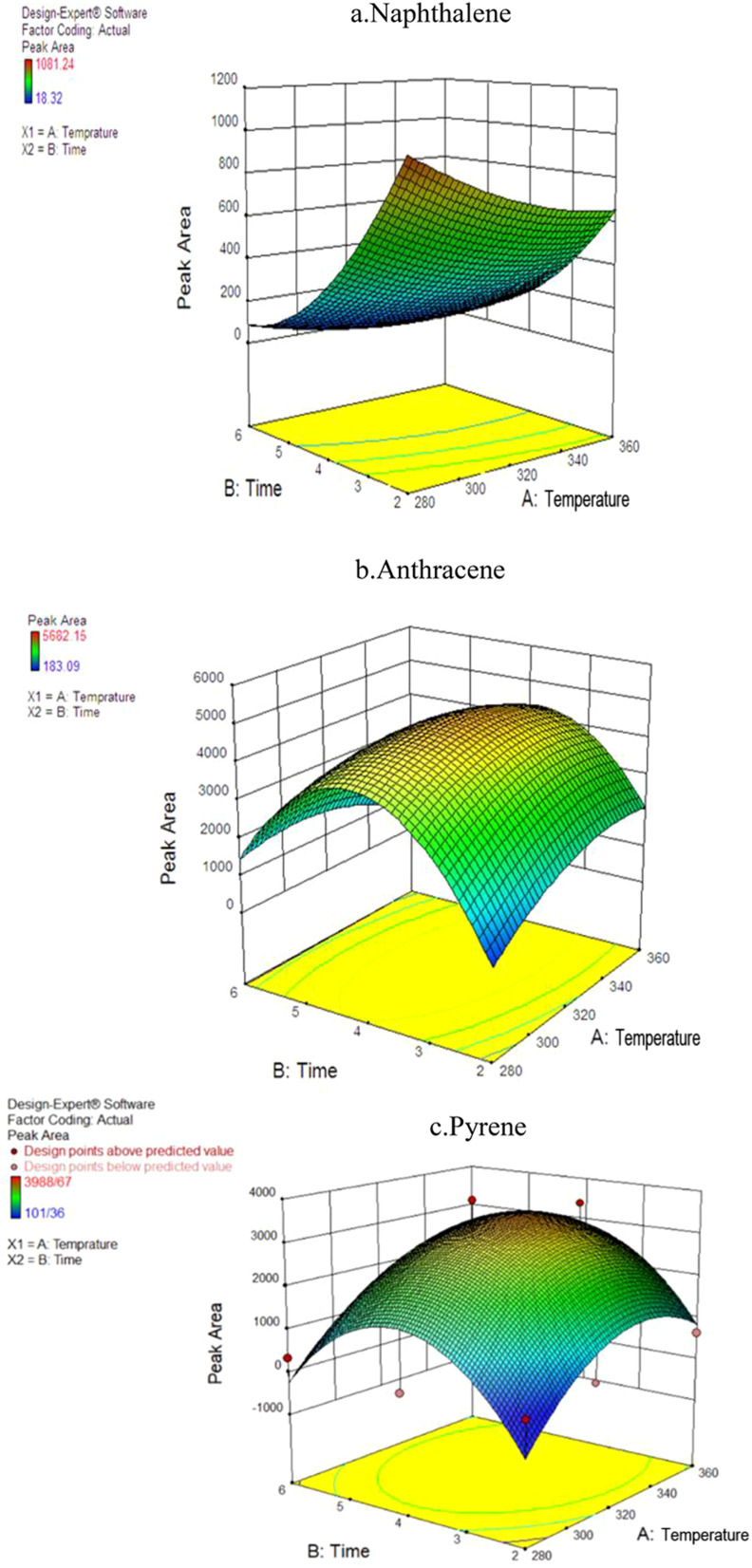
The effect of desorption variables on the efficiency of NTD-PAF-6 MNPs: (a) naphthalene, (b) anthracene, (c) pyrene.

For optimization purposes, the effects of time and temperature during the desorption of the PAH compounds were investigated using Response Surface Methodology (RSM) in the Design-Expert software (version 7.0; USA Stat-Ease). The ANOVA results indicated that the second-degree model was statistically significant, as evidenced by its low standard deviation and high *R*-value. Fig. 4 shows the simultaneous effect of temperature and length of time (during NTD injection) on the peak area of pyrene, anthracene, and naphthalene.

Interestingly, the findings differed from those of similar studies that asserting that higher temperature and longer desorption time leads to increase trap efficiency of the analyte. In this study, varying results were obtained for different analytes. The interaction between temperature and desorption time exhibited the highest peak level for the naphthalene analyte. Consequently, the optimal desorption time and temperature for NTD containing PAF-6 absorbent were determined to be 280 °C and 2 minutes, respectively. Moreover, the optimal temperature for pyrene and anthracene analytes was approximately 325 °C with an optimal injection time of 3.9 minutes. The results indicate that increasing the desorption temperature from 280 to 320 °C enhanced the chromatograms area of PAH compounds. However, consistent with the findings of Ghelichi *et al.*^34^ raising the temperature above 350 °C negatively impacted the chromatogram area of PAH compounds. This discrepancy in optimal desorption temperature is attributed to variations in the boiling points of PAHs. Furthermore, increasing the desorption time from 2 to approximately 3.9 minutes increased the desorption of NTD sample; however, further prolongation of desorption time did not enhance sampling desorption. Notably, PAF-6 offers the advantage of easy purification at a shorter duration and temperature. Nevertheless, in two similar studies conducted by Soury *et al.*^21^ and Ghalichi *et al.*^34^ on MOFs and XAD-2 polyaniline, the optimal temperature for the three studied analytes was approximately 350 °C with an optimal injection time of 7–8 min. These results suggest the desorption of compounds from the packed adsorbent was more efficient in those studies, leading to faster cleaning of the adsorbent compared to the sorbents used in this study.

### 3.3 Breakthrough volume investigation

**Fig. 5:**
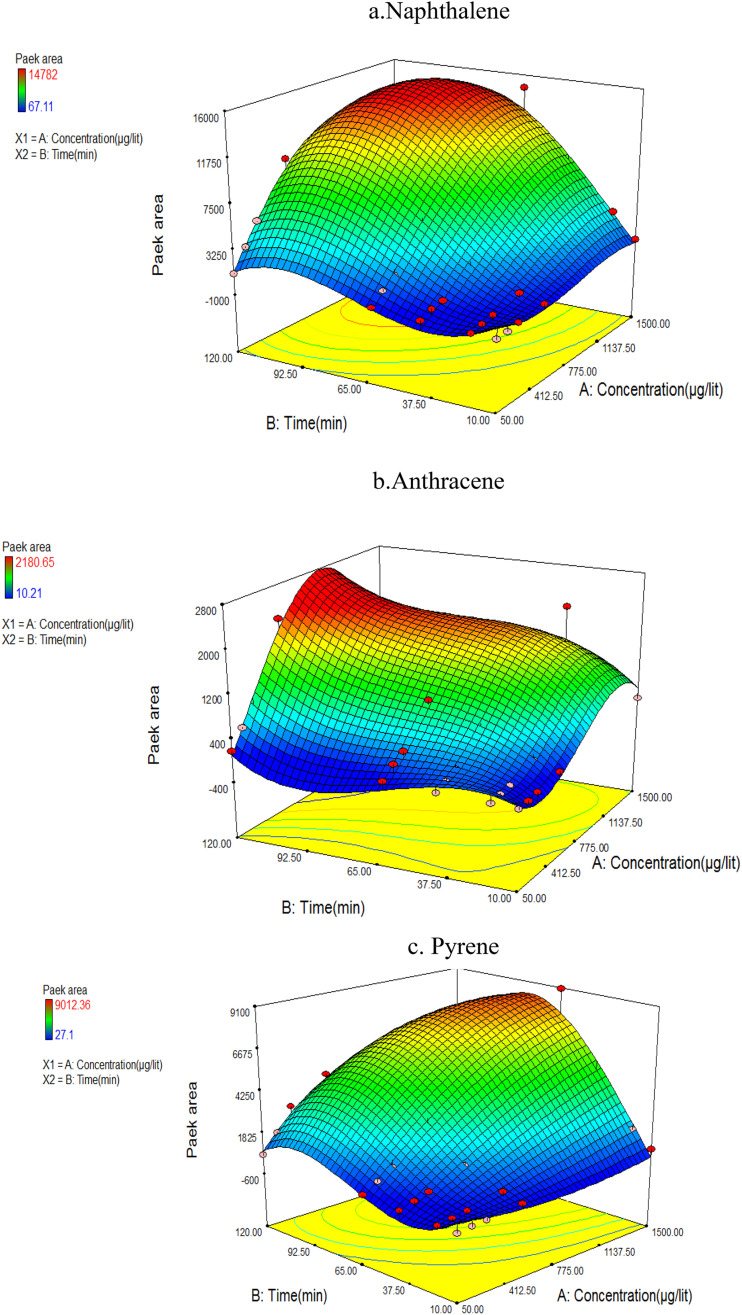
Interaction between analyte concentration and air volume sampled by the NTD-PAF-6 MNPs technique (a) naphthalene (b) anthracene (c) pyrene.

The analysis of BTV test data revealed that the concentration of compounds collected by NTD decreased with increasing sampling time, at a certain point, indicating the occurrence of a breakthrough. Fig. 5 shows the breakthrough volume of the NTD adsorbent in PAHs sampling from the air. As shown in Fig. 5, the results indicated that at PAH concentrations below 1100 μg L^−1^ in the chamber, the trapping of the analytes of interest increased within the sorbent packed inside the NTD, while at concentrations exceeding 1100 μg L^−1^ and sampling contact times surpassing 90 min resulted in decrease analytes trapping and breakthrough. The sampling PAH compounds within the concentration range of 50 to 1100 μg L^−1^ in the sampling chamber, demonstrated that the current adsorbent possesses a high absorption capacity and enabling sampling of PAH at concentrations within the permissible limits for extended durations. In a similar study, the BTV value for naphthalene was reported to be 6 times the threshold limit value (TLV) while for pyrene and anthracene it was 21 times the TLV.^34^ The BTV phenomenon is influenced by the vapour pressure of target analytes. Chemical pollutants with higher vapor pressure reach the breakthrough stage faster than others. An increase in vapour pressure and volatility will decrease the capacity of the pollutant adsorbent.^21^

### 3.4 Method validation

#### 3.4.1 Repeatability & reproducibility

The repeatability and reproducibility of the proposed sampling method were evaluated by calculating the standard deviation of the experimental results. The repeatability/reproducibility were assessed over four instances across three consecutive days and the mean values are presented in Table 1. The results demonstrate that, NTD containing PAF-6 absorbent exhibits high sensitivity in sampling and analyzing PAHs in the air. Consequently, the NTD method containing PAF-6 absorbent showcases significant repeatability and reproducibility capabilities, rendering it a valid and reliable method for analyzing such air pollutants.

**Table 1:** Data results of reproducibility evaluation tests

Analyte	Average daily repeatability (μg L^−1^)	RSD		Average daily reproducibility (μg L^−1^)	RSD
Naphthalene	0.429343	0.192	NTD1	0.474280	0.200
	0.424010		NTD2	0.394807	
	0.430837		NTD3	0.468924	
Anthracene	0.044181	0.205	NTD1	0.096444	0.218
	0.036422		NTD2	0.005643	
	0.029169		NTD3	0.046698	
Pyrene	0.224481	0.178	NTD1	0.223296	0.229
	0.212269		NTD2	0.150977	
	0.236713		NTD3	0.187384	

#### 3.4.2 Accuracy

The accuracy of the current method was evaluated through side-by-side sampling with NTD packed with PAF-6-MNPs and NIOSH-5515 methods. Samples were collected from a pilot chamber with different concentrations ranging from 0.25 to 3.0 times the TLV-TWA for naphthalene, anthracene, and pyrene. The results indicated a significant correlation between the data from both methods. The correlation coefficients for naphthalene, anthracene, and pyrene were, 0.87, 0.83 and 0.89, respectively.

#### 3.4.3 LOD, LOQ, and LDR

To assess the efficiency of NTD containing PAF-6-MNP for sampling PAHs, LOD, LOQ, and LDR were determined for naphthalene, anthracene, and pyrene analytes. The results of these tests are summarized in Table 2. The LOD value in the NIOSH-5515 method^30^ is typically reported to range from 0.3–0.5 μg L^−1^. In the study by Souri *et al.*^21^ LOD values for naphthalene, anthracene, and pyrene were reported as 0.011, 0.015, 0.01 μg L^−1^ respectively, with LOQ values of 0.04, 0.03, 0.05 μg L^−1^. In Ghelichi *et al.*'s study^34^ LOD values of 0.09 and 0.23 were reported for naphthalene. Notably, in the present study, the LOD was lower than those reported in similar previous studies, suggesting that the proposed method offered very high sensitivity for detecting the desired analytes. Furthermore, a comparison with other studies is presented in Table 3. The LOD values of PAH compounds in the conventional NIOSH method for sampling and analyzing PAHs with a sorbent tube are higher than that in the current method, indicating lower sensitivity compared to the NTD. Consequently, the NIOSH method is deemed insensitive and unsuitable for use in air environments where PAH concentrations are below 0.3 μg L^−1^. Moreover, this method requires an organic solvent during sample preparation that is toxic and can impact operators in the laboratory, while the NTD is a solvent-free method.^30^

**Table 2:** LODs, LOQs and LDR, of the PAF-6 MNPs -NTD for the determination of some PAHs in air samples

Analyte	LDR (μg L^−1^)	*R* ^2^	LOD (μg L^−1^)	(LOQ (μg L^−1^)
Naphthalene	0.000015–205	0.96	0.0051	0.0150
Anthracene	0.00001–1.18	0.98	0.0034	0.0104
Pyrene	0.00012–1.34	0.97	0.0041	0.0125

**Table 3:** Comparison of the proposed NTD with other available method for sampling of PAHs

Method	Sorbent	LOD	LOQ
NIOSH-GC^30^	XAD-2	0.3–0.5 (μg L^−1^)	—
OSHA-HPLC^33^	XAD-2	0.007–0.8 (μg L^−1^)	0.023–2.6 (μg L^−1^)
DI-CF-SPME-GC/MS^35^	PDMS	0.01–0.05 (μg L^−1^)	0.02–0.15 (μg L^−1^)
NTD-GC-FID^36^	Cot/Go/Si nanocomposite	0.057–0.17 (ng g^−1^)	0.23–0.65 (ng g^−1^)
CA-INCAT-GC-FID^37^	PANI/MWCNT	0.0028–0.025 (ng g^−1^)	—
NTD-GC-FID^38^	TiO2 nanotube	0.026–0.82 (μg L^−1^)	—
NTD-GC-FID^34^	XAD-2/PANI	0.002–0.09 (ng L^−1^)	0.01–0.23
NTD-GC-FID^20^	Zn (MOFs)	0.01–0.021 (μg L^−1^)	0.03–0.07 (μg L^−1^)
NTD-GC-FID (current research)	PAF-6-MNPs	0.0034–0.0051 (μg L^−1^)	0.010–0.015 (μg L^−1^)

### 3.5 Storage time

**Fig. 6:**
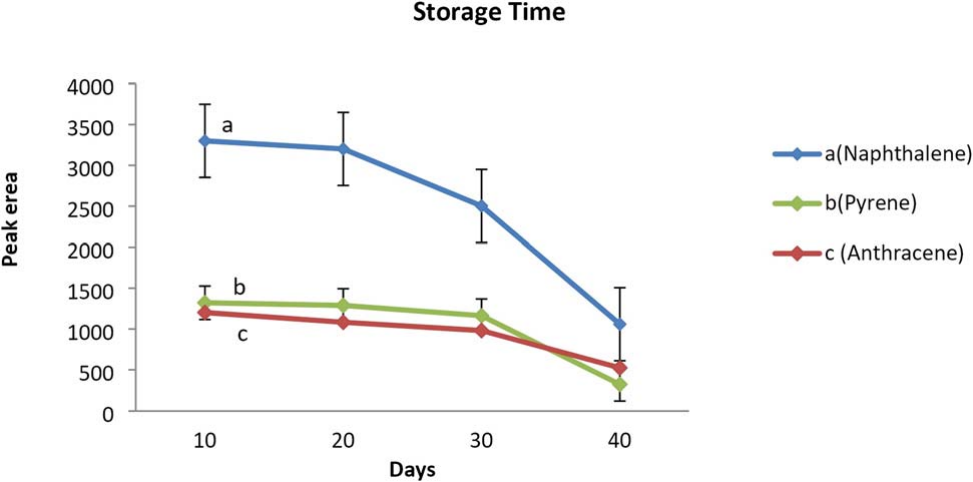
Storage stability of the NTD packed with PAF-6-MNPs during the 40 day period at temperature of 4 °C. (a) Naphthalene, (b) pyrene, (c) anthracene.

Analyte storage capacity of the sorbent packed inside the proposed NTD was evaluated by analyzing the samples taken immediately, as well as 10, 20, and 30 days after sampling. The average peak area responses corresponding to each analyte of interest were compared with the control sample which was immediately analyzed on the first day of sampling. The results revealed that the amount of analytes in the samples did not significantly decrease compared to the initial amount, even after 20 days of sampling. However, the amount of analytes adsorbed on sorbent packed inside the NTD decreased significantly after 30 days of sampling compared to the initial amount. Therefore, it can be concluded that the proposed NTD packed with the PAF-6-MNPs sorbent, when evaluated after sampling PAHs can be stored in the refrigerator (4 °C) for up to 20 days. It is noteworthy that in the NIOSH-5515 method,^30^ the storage capability of the XAD2 sorbent tube has not been reported for such analytes (Fig. 6). Similar studies, have shown that the storage capability of the NTD sampler containing different adsorbents in PAHs sampling aligns with the above results.^21^

### 3.6 In-field measurements

After optimizing various parameters affecting the performance of the proposed NTD, its performance was compared with the gas-phase outlined in the NIOSH-5515 standard (considered as the gold standard).^30^ Subsequently the packed, NTD with PAF-6-MNPs employed for sampling PAHs in the exhaust gas of diesel cars in-field sampling. Next, the packed NTD with a flow rate of 0.5–0.8 mL min^−1^ was used for 30 min of sampling at a specific distance from a diesel car's exhaust. Side-by-side sampling was conducted with the NIOSH 5515 method^30^ (with a sampling flow rate of 120 mL min^−1^ for 30 min). The results of the side-by-side sampling with both methods indicated that the NIOSH-recommended method^30^ was unable to detect the levels of naphthalene, anthracene, and pyrene in the exhaust gas of diesel cars. Conversely, in the same samples taken with the proposed NTD, concentrations of anthracene and pyrene were measured to be 0.0003 and 0.0187 mg m^−3^, respectively. These concentrations of PAH compounds from diesel car exhaust were below 0.1 μg mL^−1^ (LOD of the NIOSH method), which were undetectable by the NIOSH method.

However, there were some limitations in conducting this study. For instance, in laboratory conditions, it was not feasible to prepare PAHs compounds in the particle phase, so the PAHs compounds were solely sampled and analyzed in the gas phase.

## 4. Conclusions

In the present study, PAF-6 MNPs were synthesized and modified within the laboratory setting, subsequently integrated into the NTD sampler for the inaugural sampling and analysis of PAHs in air. The method underwent optimization in the laboratory and demonstrated acceptable performance in sampling PAHs in the air. This method proved capable of detecting PAH compounds at low concentrations without necessitating sample preparation or chemical compounds. Additionally, through heating in the injection port of GC, pollutants are transferred from the NTD into column of the gas chromatography, and enabling the reuse of the adsorbent. The breakthrough of samples depends on concentration, and the sorbent exhibits a significant capacity for sampling of PAH in ambient air. The maximum storage time for PAHs samples with the proposed NTD was determined to be 20 days. A comparison of the results obtained from the NIOSH and NTD methods revealed the high reproducibility and sensitivity of the NTD method for sampling and analysis of PAHs.

## Conflicts of interest

The authors declared that there were no competing interests regarding this paper.

## Supplementary Material
