# Correction: Development of a needle trap device packed with modified PAF-6-MNPs for sampling and analysis of polycyclic aromatic compounds in air

**DOI:** 10.1039/d4ra90074j

**Published:** 2024-07-08

**Authors:** Mobina Hashemi, Abdulrahman Bahrami, Farshid Ghorbani-Shahna, Abbas Afkhami, Maryam Farhadian, Ali Poormohamadi

**Affiliations:** a Center of Excellence for Occupational Health, Occupational Health and Safety Research Center, School of Public Health, Hamadan University of Medical Sciences Hamadan Iran bahrami@umsha.ac.ir; b Department of Analytical Chemistry, Faculty of Chemistry and Petroleum Sciences, Bu-Ali Sina University Hamedan Iran; c Department of Biostatistics, School of Public Health and Research Center for Health Sciences, Hamadan University of Medical Sciences Hamadan Iran

## Abstract

Correction for ‘Development of a needle trap device packed with modified PAF-6-MNPs for sampling and analysis of polycyclic aromatic compounds in air’ by Mobina Hashemi *et al.*, *RSC Adv.*, 2024, **14**, 18588–18598, https://doi.org/10.1039/D4RA01651C.

The authors regret that the name of one of the authors (Abbas Afkhami) and one of the affiliations (affiliation *b*) were shown incorrectly in the original article. The corrected author list and list of affiliations are as shown herein.

The Royal Society of Chemistry apologises for these errors and any consequent inconvenience to authors and readers.

